# Supplementary material for: Are carbon emissions trading and green financial instruments synergistic? -Comprehensive quantitative research based on content analysis
Source: PLoS One. 2024 Mar 7;19(3):e0298601. doi: 10.1371/journal.pone.0298601 (PMC10919601; doi:10.1371/journal.pone.0298601)
Supplement: S1 Data — (ZIP) [file pone.0298601.s001.zip › Data/地级市Z_F工作报告.zip!/╕ú╜¿/╒─╓▌╩╨/└┤╫╘╣1⁄2╓┌║┼í╛┬φ┐╦╩2╛▌═°í┐_2018.docx]

各位代表：

现在，我代表漳州市人民政府，向大会报告政府工作，请予审议，并请市政协委员和其他列席人员提出意见。

一、2017年工作回顾

过去的一年，在以习近平同志为核心的党中央坚强领导下，在习近平新时代中国特色社会主义思想的科学指引下，我市各级政府认真做好迎接和学习宣传贯彻党的十九大各项工作，深入贯彻落实习近平总书记系列重要讲话精神和对福建工作的重要批示指示精神，坚决执行中央、省和市委的各项决策部署，坚持稳中求进工作总基调，坚持新发展理念，坚持以供给侧结构性改革为主线，扎实开展“三抓三比、十项竞赛”，全面推行“生态+”模式，富美新漳州建设取得新成效，市第十六届人大一次会议确定的各项目标任务全面完成。

全市完成地区生产总值3563.48亿元，增长9.1%；一般公共预算总收入318.08亿元，增长10.6%，地方一般公共预算收入204.04亿元，增长8.7%；规模工业增加值1621.31亿元，增长8.7%；固定资产投资3328.1亿元，增长17.7%；外贸出口512.6亿元，增长7.1%；实际利用外资（验资口径）12.17亿美元，增长4.6%；社会消费品零售总额982.43亿元，增长12.2%；居民消费价格总水平上涨1.1%；城镇居民人均可支配收入33359元，增长8.6%；农村居民人均可支配收入16676元，增长8.9%；城镇登记失业率2.23%；人口自然增长率12.2‰。年度节能减排降碳任务全面完成。主要工作和成效是：

（一）竞赛促比拼，经济保持较快增长。以迎接党的十九大召开为动力，认真开展“三抓三比、十项竞赛”，推动经济保持稳中有进、提质提效的良好态势，GDP增幅连续四年保持全省第一，规模工业增加值增速位居全省第一。突出项目带动，全年落实“五个一批”项目2625个，新开工亿元以上项目260个、建成或部分建成150个，金龙汽车、中海油LNG、古雷石化炼化一体化等重大项目相继落地开工。腾龙翔鹭项目重组方案获省委省政府批准，PTA项目整改修复工程有序有效推进,古雷石化园区获评“中国化工潜力园区十强”。坚持“一把手”抓招商，全市签约项目362个，计划总投资1507亿元，已落地175个。加强基础设施建设，福厦（漳）高铁、云平高速、漳武高速（南靖段）正式开工，沿海大通道（漳浦段）建成通车，完成交通基础设施投资190亿元，创历史新高。县域经济实力得到增强，龙海入选全省县域经济实力“十强”县（市），云霄、诏安、长泰、东山入选全省县域经济发展“十佳”县（市）。

（二）发力供给侧，转型升级步伐加快。深化“追问题、解难题、促发展”和“政策不睡觉、落地见成效”活动，推动产业加快转型升级。明确重视工业的发展导向，坚持一季一主题抓工业生产调度，出台系列政策措施，全力扶持实体经济渡难关、促生产，发放工业企业扶持资金1.3亿元，为155家工业企业补办不动产权登记证；新增、新上规模工业企业166家、高新技术企业20家、科技小巨人50家，完成技改投资680亿元，3项成果获省科技进步一等奖。推动现代服务业提速发展，三产增速连续三年位居全省第一；新增6个省级现代服务业集聚区，占全省1/3;设立中小企业贷款风险补偿基金，金融机构各项贷款余额增长14.6%,新增上市企业2家，实现民企A股主板上市“零”的突破;入选中国“电子商务百佳城市”，诏安、平和入选国家级电子商务进农村综合示范县；全力打造全域旅游，成功举办“花样漳州”十佳系列活动,南靖土楼荣获“中国最佳乡村旅游项目奖”，片仔癀中药工业园荣获首批“国家工业旅游示范基地”称号，旅游业接待游客人数、旅游收入分别增长22.9%和30.9%。现代农业加快发展，获批国家农业可持续发展试验示范区、农业绿色发展试点先行区，新增设施农业2.8万亩,新建现代化粮食储备仓容35万吨，11个产品入选全国名优新农产品名录。龙海荣获“中国休闲食品名城”称号。

（三）践行“生态+”，美丽漳州魅力彰显。实施中心城区“五湖四海”、南山水岸、“双百”绿化工程、圆山林下生态园等一批“生态+”先行示范项目，“一城花海半城湖”独特风光初步形成，从“生态佳”迈向“生态+”赢得广泛认可。实施规划提升工程，提请市人大常委会作出决定，将5290亩土地列为中心城区重要生态空间保护范围;编制完成5个城市设计（覆盖面积60平方公里）、6项专项规划、2个省级“多规合一”试点。开展新一批市政线网“六千工程”，建成城区停车楼（场）38个。基本建成博物馆、艺术馆、规划展示馆，圆山文化中心基本形成。配合市人大制定并颁布实施首部实体性法规《市容和环境卫生“门前三包”责任区管理若干规定》，市区生活垃圾清洁直运全面开展。完成拆违面积超700万平方米。加快厦漳同城化先行示范区建设，65个同城化项目进展顺利。建立生态文明指标考核体系，开展“三铁治污、提升三质”整治行动、城乡生态环境提升行动、大气污染整治百日攻坚，全面落实“河长制”，全力整改中央、省环保督察反馈问题，“三质”水平持续好转。完成国土绿化20万亩、水土流失综合治理30.2万亩。蝉联第五届全国文明城市称号，漳州古城保护建设（一期）项目荣获“2017年中国人居环境范例奖”。

（四）强力推改革，机制创新富有成效。持续深化简政放权，年度各项改革任务如期完成，构建开放型经济新体制综合试点试验工作取得突破。“会审制”改革、商务110、国地税联合办税、“即报即放”出口食品检验检疫方式等经验做法在全国推广。在全国首创食品农产品检验检疫“四检合一”，台商投资区保税物流中心(B型)正式获批设立。成立供给侧改革服务中心、台胞台商服务中心，营商环境持续优化，荣获“2017中国十佳最具投资营商价值城市”称号。对台对外合作成效彰显，实际利用台资34亿元；与东盟贸易额达80亿元。

（五）全力补短板，群众获得感持续提升。20项为民办实事项目基本完成，“四难一差”问题逐步缓解，“十大惠民特色工程包”完成投资190亿元。开展精准脱贫攻坚竞赛，推广平和“养鸡生蛋”工程，实现低保线与扶贫线“两线合一”，2.28万人、79个贫困村实现脱贫，完成造福工程搬迁3642户、1.2万人。启动“安得广”惠民安居工程，新建保障性安居工程1.5万套。出台城市既有住宅增设电梯指导意见和补助政策；解决历史遗留办证难问题，补办房地产不动产权证4814份。

拓宽就业渠道，新增城镇就业4.83万人。加快医疗卫生事业改革发展，公立医院综合改革成效显现，新增床位1031张、卫技人员1089名。教育事业不断进步，新建57所公办幼儿园，新增幼儿园学位1.34万个、中小学学位6989个。体育事业发展加快，成功举办第十三届市运会、第十届老健会，新增3个国家高水平体育后备人才基地。文化事业繁荣发展，《漳州文库》编撰进展顺利，长篇报告文学《谷文昌》获评中宣部“五个一工程奖”。深化平安漳州、法治漳州建设，开展“护航金砖、共筑平安”四项行动、维稳“六大专项行动”，社会保持安定稳定。推进安全生产大排查大整治百日行动，全面做好国务院、省政府安委会安全生产巡查问题整改。深入开展“食品安全放心市”创建工作。建成全省唯一的全国示范数字档案馆。外事侨务、民族宗教、苏区老区、山海协作、防震减灾、援藏援疆援宁等工作稳步开展。

（六）扎实改作风，自身建设不断加强。坚定不移推进全面从严治党，牢固树立“四个意识”，严格执行《准则》、《条例》，扎实推进“两学一做”学习教育常态化制度化。认真落实中央八项规定及实施细则，坚决反对“四风”，加强机关效能建设，大力整治“庸懒散拖”，政风行风持续改善。市本级“三公”经费支出下降35.4%。在市委“一线考察干部工作法”引导下，干部干事创业精气神明显提升。认真执行人大决定决议，自觉接受人大、政协和社会监督，办理人大代表建议198件、政协委员提案287件，办复率100%。各民主党派参政议政、民主监督富有成效。政务“五公开”持续推进，行政监察、审计监督继续加强，党风廉政建设和反腐败工作有效推进。

各位代表，成绩的取得来之不易，这是党中央、国务院和省委省政府、市委坚强领导的结果，是全市人民齐心协力、团结奋斗和各方面大力支持的结果。在此，我代表市人民政府，向全市人民，向人大代表、政协委员、各民主党派、工商联、各人民团体、离退休老同志和各界人士，向驻漳省部属单位、驻漳部队、武警官兵，企业界人士和来漳创业务工人员，向关心支持漳州发展的港澳台同胞、海外乡亲和国际友人，表示衷心的感谢！

同时，我们也清醒地认识到发展中还存在不少困难和问题，主要是：经济总量仍然较小，人均水平偏低，产业发展水平不高、结构不优，创新能力不足；城乡区域发展不均衡，城镇化率偏低，生态环境保护压力较大；优质教育、医疗、养老资源不足，基本公共服务存在不少短板；体制机制创新和政府职能转变还不到位，发展环境仍需优化等等。我们必须直面问题，创新举措，立项挂牌督办，切实加以解决。

二、以党的十九大精神为指引，扎实推进2018年工作

党的十九大作出了中国特色社会主义进入新时代的重大政治论断，确立了习近平新时代中国特色社会主义思想，深刻阐述了新时代中国共产党的历史使命，确定了决胜全面建成小康社会、开启全面建设社会主义现代化国家新征程的目标，对新时代推进中国特色社会主义伟大事业和党的建设新的伟大工程作出了全面部署。学习宣传贯彻党的十九大精神是当前和今后一个时期的首要政治任务。

今年政府工作的总体要求是：全面贯彻党的十九大精神，以习近平新时代中国特色社会主义思想为指导，认真落实中央经济工作会议、省委十届五次全会和市委十一届六次全会部署，坚持党对经济工作的领导，坚持稳中求进工作总基调，坚持新发展理念，紧扣社会主要矛盾变化，按照高质量发展的要求，统筹推进“五位一体”总体布局和协调推进“四个全面”战略布局，坚持以供给侧结构性改革为主线，把高质量发展与实现赶超有机统一起来，持续深化“三抓三比、十项竞赛”，抓牢抓实增长点，奋力赶超作贡献，一二三产齐发力，重中之重抓古雷，推动质量变革、效率变革、动力变革，坚决打好三大攻坚战，促进经济社会持续健康发展，推动富美新漳州建设迈出新步伐。

经济社会发展主要预期目标是：地区生产总值增长9.5%左右；一般公共预算总收入增长6%左右，地方一般公共预算收入增长6%左右；固定资产投资增长16%左右；规模工业增加值增长9%左右，规模工业总产值增长9.3%左右；外贸出口增长3%左右；实际利用外资增长3%左右；社会消费品零售总额增长11%左右，居民消费价格总水平涨幅控制在3%左右；城镇登记失业率控制在3.6%以内；城镇和农村居民人均可支配收入分别增长9.5%左右；做好“十三五”规划中期评估工作；落实节能减排降碳任务。

实现上述目标，关键要做好以下六个方面的工作：

（一）突出实体经济，推动高质量发展

加快发展先进制造业。实施产业龙头促进计划和新兴产业倍增计划，分行业出台扶持政策，力争石化产业产值400亿元以上、食品产业产值2000亿元以上、装备制造产业产值1200亿元以上、钢铁及钢铁制品产业产值500亿元以上，电子信息、新能源、生物医药等战略性新兴产业产值合计400亿元以上。实施“双高”培育工程，建立“独角兽”种子企业储备库并加以扶持。实施重点企业倍增计划，用3年左右时间，推动试点企业规模倍增、效益倍增、纳税倍增。培育壮大57家龙头企业、126家高成长企业，新增、新上规模工业企业100家以上，开工建设三宝耐腐蚀热轧卷板、福欣节镍不锈钢深加工、猛狮锂离子电池二期三期等一批扩量提质项目。创建食品、钟表产业发展基金，加快建设食品总部产业园、时间（钟表）小镇，培育一批食品、钟表航母企业。创建国家和省级智能制造、数字化车间试点，实施“数控一代”、“智能一代”应用示范工程，提高制造业数字化、智能化水平。落实工业技改奖励政策，实施省级重点技改项目100个以上，技改投资增长15%。

大力培育新增长点。实施“五新”工程，推动新产业、新技术、新平台、新业态、新模式与实体经济深度融合，在中高端消费、创新引领、绿色低碳、共享经济、现代供应链、人力资本服务等领域培育新增长点、形成新动能。推进华为云计算数据中心、敦煌网等项目建设，推动制造业加速向数据化、网络化、智能化发展，培育壮大数据经济。合理开发利用海洋资源，加快发展海洋优势产业。扶持发展建筑业，培育一批创新能力强、机械化水平高的建筑业企业。

集中力量发展重点区域。高质量、高标准推进重点区域率先突破，聚力做强若干经济增长极。重中之重抓古雷，更加注重环保、安全、质量和效益，着力完善公共服务配套，加快建设古雷石化炼化一体化项目，推动PX、海顺德特种油品等项目重新生产，促进一批芳烃下游产业项目开工投产，加大中下游石化产业链招商，加快实现基地化布局和全产业链发展。厦漳同城化先行示范区要促先行，全面对标、对接厦门，增强城市和产业支撑，加快推进金龙汽车、惠尔康生物科技、芯云谷、泰禾国际医院等项目，支持龙海建设南太武南溪湾片区，确保增速高于全市平均水平1-1.5个百分点。中心城区要当中坚，放大生态优势，发展高端高质高新产业和现代服务业，加快提升城市魅力、壮大城市经济。各县（市、区）要明确各自重点区域，依托特色资源，集中突破1-2个重点优势产业，发展壮大县域经济。坚持生态优先、产城融合，设立工业园区发展基金，推动开发区新一轮创业，做强“一县一园区”。

推动服务业优质高效发展。主动适应需求变化，创新培育服务业新业态、新形态、新模式、新主体，加快打造发展新引擎。引导制造业主辅分离，加快发展工业设计、信息服务、会展服务等服务外包产业，带动制造业企业向服务型制造转型。提升现代物流、电子商务、商贸流通效率，推进专业市场现代化改造提升，加快发展智慧物流、冷链物流和第三方、第四方物流。大力推进全域旅游发展，实施三年行动计划，进一步完善公共配套，策划旅游套餐，打造精品线路，打响“清新福建、花样漳州”品牌。围绕打造全国知名的“颐养之城”，加快发展健康养生产业，打造一批以生态养生为特色的健康服务示范区。

（二）优化供给质量，推动增长动力转换

优选增量，扩大有效投资。发挥投资对优化供给结构的关键性作用，实施“十百千支撑工程”，突出基础设施、产业、社会民生项目，安排重点项目561个、年度投资1308亿元，新开工200个，建成或部分建成150个。提高产业投资比重，建设投资500万元以上工业项目1000个，开工亿元以上工业项目300个、投产300个。推进补短板工程包，完成“十大特色惠民工程包”投资150亿元以上。打好交通项目建设攻坚战，实施24个重点攻坚项目，推进城市轨道交通、沿海高铁（漳州-汕头段）前期工作，加快福厦（漳）高铁、漳武高速（南靖段）和14个国省干线互联互通项目建设，确保厦漳同城大道、厦蓉高速改扩建工程、九龙岭隧道改造工程建成通车，着力解决交通网络瓶颈问题。出台激发民间有效投资扶持政策，对医疗、养老、教育等领域市场化项目实行“不再审批”模式，新对接民企合同项目投资500亿元以上。

创新引领，加速动力变革。完善创新生态体系，统筹推进科技链、产业链、金融链“三链融合”，培育一批具有创新能力的排头兵企业，新增高新技术企业和省级创新型企业25家。整合提升创新平台，加快推进猛狮锂电池研究院、正兴汽车轻量化研究院、立达信科技小镇等项目建设。健全科技成果转化激励机制，实施大中型工业企业研发机构广覆盖行动。落实好《关于促进人才优先发展的若干措施》，加大两院院士、“千人计划”专家、高层次医疗专家等高端人才的柔性引进力度，成立人才发展集团，创造条件吸引人才、留住人才。

降本增效，提高供给效率。突出“破、立、降”，落实“去降补”年度任务，提高供给质量和效率。出台一批稳增长政策，加大涉企税费清理，大力降低实体经济成本。稳妥处置“僵尸企业”，盘活低效用地，倒逼落后产能退出、低效企业转型。关停淘汰落后燃煤小锅炉，推动集中供热。创新破解用地难、用工难等要素瓶颈，加快推进工业企业补办不动产证工作。支持民营企业发展，落实保护产权政策，加强企业家队伍建设，激发和保护企业家精神。

（三）立足生态优势，更高水平建设美丽漳州

以“生态+”引领城市发展。围绕率先建成国家生态文明试验区目标，加强生态保护、生态修复、生态提升，持续深化“生态+”模式，延展“生态+”效益，推动“生态+”向城市、产业、园区、旅游等全领域嵌入，努力实现生态环境的“高颜值”和经济发展的“高素质”。集成打造绿城、花城、水城、历史文化名城，又好又快推进“五湖四海”、“芗江水岸、龙江观竹”生态项目、达达通绿道等建设，有机串联江、湖、山、园、城，形成“一溪贯五湖四海，两山接千年古城”生态空间格局。推动各地建设一批“生态+”示范项目，带动全市形成“城中有湖、城中有林、城中有花”。

以功能配套提升宜居水平。强化规划执行力，完成《漳州市城市总体规划（2012-2030年）》修改，加快推进“多规合一”、城市“双修”、城市设计。实施民生基础设施建设九大工程，重点在治堵、治涝、治污上下功夫，完成投资560亿元。开展市区交通畅通工程，新建改造城市道路150公里，新增市区公共停车泊位850个，加快形成“一环”城市交通网。统筹推进重点片区开发，全面开展西湖生态园配套建设，大力实施老旧小区、农贸市场改造提升。推进海绵城市建设，开展易涝点隐患排查整治。开工建设市区第二水源。新（扩）建5座垃圾焚烧发电厂。推进“厕所革命”，新建221座城乡公厕、新改扩建 56座旅游厕所。深化城管执法体制改革，开展新一轮“两违”综合治理。巩固全国文明城市创建成果，全面落实“门前三包”，提升城市精细化管理水平。

以铁的决心打好污染防治攻坚战。深化“三铁治污、提升三质”整治行动，开展“清新水域、洁净蓝天、清洁土壤”三大工程，确保主要污染物排放总量明显减少。重中之重打赢蓝天保卫战，突出臭氧污染治理，开展挥发性有机物、建筑施工及道路扬尘专项整治，确保空气质量有效提升。加强重点流域和近岸海域污染治理，中心城区基本消除黑臭水体，其他小流域消除劣V类水体、消灭“牛奶溪”，持续改善水环境。开展土壤污染治理与修复，加强重金属污染防治，提高危险废弃物处置能力。加大“五海”资源保护。加强生态系统保护，完成国土绿化10.2万亩、水土流失综合治理18万亩。深化行业污染治理，加大工业污染减排力度。推进第二次全国污染源普查。

以人为核心加快城镇化步伐。加速中心城区全域城市化，有序推进镇改街、村改居。加快发展城市经济，创造更多就业岗位，增强对农业转移人口的吸引力和承载力。实施“大县城”战略，推进县城提质扩容，引导特色小镇健康发展，增强产业发展能力和交通节点功能，提高户籍人口城镇化率。建立健全城乡融合发展的体制机制和政策体系，清除阻碍要素下乡各种障碍，加快实现基本公共服务均等化。

（四）实施乡村振兴战略，推动农业农村优先发展

整合提效益，让农业更强。制定乡村振兴战略规划，深化农业供给侧结构性改革，加大产业、资源、要素、资金、人才、品牌的整合，打造全产业链产值均超百亿元的九大优势特色产业。全域建设国家农业可持续发展试验示范区和农业绿色发展试点先行区，加快质量兴农、绿色兴农步伐。补充耕地0.8万亩，完成粮食总产量67万吨以上，确保粮食安全。扶持“两朵花、三泡茶、四珍菌、六条鱼、十大果”，新增、续展“三品一标”50个，创建一批国内有影响力的农产品品牌。发挥农业园区辐射带动作用，创建国家级现代农业产业园1个、省级10个，新增设施农业2万亩。做大农产品精深加工业、休闲农业等新业态，建立农产品产销联盟，推进一二三产融合发展。狠抓农产品质量安全监管，实施“五个专项行动”，创建国家级出口食品农产品质量安全示范市。

持续扩投入，让农村更美。实施基础设施惠民工程，完善农村供水、供电、通信等基础设施，建好管好护好运营好农村公路。优先发展农村教育事业，完善农村社会保障体系，推进健康乡村建设。重视农村建房规划管理，加快农村裸房整治和危房改造，保护好历史文化名镇、名村、传统村落。支持有条件的乡村建设田园综合体。推进农村建设“双百”行动、农村污水垃圾治理三年攻坚行动，实现农村污水处理、农村环境卫生保洁制度全覆盖，建设更多美丽乡村。深化农垦国有农场、国有林场和农村集体产权制度改革，确保集体资产保值增值。健全自治、法治、德治相结合的乡村治理体系，推进移风易俗，培育文明乡风、良好家风、淳朴民风，促进乡村文化兴盛。

全力促增收，让农民更富。更加关注农民、依靠农民，让农民有活干、有钱赚。落实农村土地“三权”分置，完成土地承包经营权确权登记颁证，拓宽农民财产性收入。积极培育职业农民，壮大新型农业经营主体，促进小农户和现代农业发展有机衔接。推进农村金融服务创新，加强“三农”保险基层服务体系建设，推动各类政策、项目、资金向强农富农领域集聚。出台政策扶持农村电商发展，加快建设农产品电商平台，培养市场经纪人、电商带头人，带动农民增产增收。

（五）深化改革开放，释放机制创新红利

试点带动，深化改革攻坚。大力推进构建全国开放型经济新体制综合试点试验工作，推动形成更多漳州经验和模式。以“一趟不用跑”和“最多跑一趟”深化“放管服”改革，对标国内外先进地区，进一步规范事项管理，推进行政服务中心标准化工作，开工建设新市行政服务中心。打好防范化解重大风险攻坚战，落实金融工作三大任务，提高直接融资比例，大力发展普惠金融、绿色金融、科技金融，提高金融服务实体经济能力；坚决打击违法违规金融活动，有效压降不良贷款，规范地方政府举债行为，营造良好金融生态。深化商事制度改革，加快推进“多证合一、证照分离”。继续深化事业单位改革。加强国有资产监管，支持国有企业做大经营性资产。

深化合作，拓展开放空间。强化“一把手”抓招商，建立专业招商队伍，重视商会招商、以商引商，继续实行招商引资、项目对接考核，制定第三方招商奖励政策，新签约“三维”合同项目250个以上、总投资1000亿元以上。注重提高出口质量和附加值，创新“漳州味·世界行”运行模式，加强与“一带一路”沿线国家和地区的双向投资与贸易合作。加快口岸建设，促进口岸开放，提高通关便利化水平。传承月港辉煌，弘扬漳州海商精神，加强“海丝”遗址保护和申遗工作，做大“海丝”文化品牌。加强与新生代海外乡亲交往，紧密联系更多港澳侨乡亲，进一步凝聚侨心、发挥侨力。

深度融合，提升对台优势。秉承“两岸一家亲”理念，实施“四个一”工程，努力在漳台融合发展上有新作为、新成效。以产业合作为抓手推动经济融合，密切与岛内工商团体联系，加大石化、精密机械、光电、食品加工等先进制造业以及文化创意、医疗养老等新兴服务业的对接合作。更好发挥台商台胞服务中心作用，支持在漳台企转型升级，加快台湾青年创业基地建设，维护台商台胞合法权益。办好“农博会·花博会”、海峡论坛相关活动，加强祖地文化、宗教文化和宗亲交流，吸引更多台胞融入漳州、扎根漳州。

（六）加速补齐短板，共建共享幸福漳州

打好精准脱贫攻坚战。把提高脱贫质量放在首位，扎实推进脱贫攻坚战三年行动，确保全市3个重点县和最后的50个贫困村、4419个农村贫困人口全部脱贫摘帽。注重扶贫同扶志、扶智相结合，精准落实产业、就业、住房、金融、医疗、教育、低保兜底等扶贫措施。实施“三千”工程，助推精准脱贫。整合各类扶贫资金，创新“养鸡生蛋”扶贫脱贫长效机制，建立贫困村、贫困户资产性增收长效机制。强化脱贫攻坚责任和监督，开展扶贫领域腐败和作风问题专项治理。落实后续脱贫措施，构建稳定脱贫、有序退出机制，确保“脱真贫、真脱贫”。

扩大优质公共服务供给。优先发展教育事业，新配扩建公办幼儿园62所，新增学位1.5万个，全市普惠性幼儿园在园幼儿占比78%以上，所有民办幼儿园纳入统一监管；实施城镇中小学扩容和义务教育提升工程，开工建设漳州一中新高中部、职业教育园区，支持高职院校扩容建设，促进和规范民办教育发展；深化教育体制机制改革，加强教师队伍建设，提高教学质量水平。加快健康漳州建设，开工建设市医院高新区院区，确保市急救中心建成完工、市公共卫生服务中心投入使用，新增床位1200张、卫技人员1900名；开展基层医疗卫生服务能力提升三年行动，加强儿科、产科、精神科和临床重点专科建设；深化医药卫生体制改革，加快建立现代医院管理制度，建成2个以上紧密型医联体，传承发展中医药事业。加快国家级医养结合试点城市建设，新建15个居家社区养老服务中心、150个农村幸福院，新增养老床位2500张。完善公共文化服务体系，丰富群众性文化生活；保护传承优秀地方文化，推进文艺精品创作，推动传统手工艺与现代文创产业融合发展。广泛开展全民健身活动，完善四级全民健身网络，建设生态体育公园、全民健身综合体。重视计生工作，促进人口均衡发展。加快推进市、县综合档案馆建设。做好二轮志书送审验收工作。

完善社会保障体系。实施25项为民办实事项目。促进高校毕业生、失业青年、失地农民等重点群体就业，新增城镇就业4.4万人。全面实施全民参保计划，完善城镇职工、城乡居民基本养老保险制度。坚持房子是用来住的、不是用来炒的定位，加快建立多主体供给、多渠道保障、租购并举的住房制度和稳定房地产长效机制，促进房地产市场平稳健康发展。加快解决商品房不动产证办理遗留问题。开工建设“安得广”惠民安居首期工程，完成棚户区改造9640户，新建保障性安居工程9943套。完善社会救助制度，保障妇女儿童、青少年、老年人合法权益，关爱农村留守儿童健康成长。

加强民主法治建设。深化法治漳州、法治政府建设，加大全民普法力度，认真做好法规草案的起草工作，适时向市人大常委会提出议案。出台法规草案和政府规章制定程序规定，提高政府立法质量和效率。加强规范性文件备案审查和重大行政决策合法性审查，做到有件必备、有备必审、有错必纠。强化执法监督，严格规范公正文明执法。推进诚信体系建设，完善守信激励和失信惩戒机制。自觉接受人大及其常委会监督、政协民主监督、社会和舆论监督，提高建议提案办理实效。

提高社会治理水平。深化平安漳州建设，常态化落实“五级责任链条”工作机制，及时化解社会矛盾纠纷。持续开展暴力、侵财、金融、网络、毒品等各类违法犯罪打击行动和交通、消防、枪爆、食药环、制售假整治行动，全面净化社会治安环境。深化“12345”服务平台运用，加快“雪亮工程”建设，提升分类处置和打防管控水平。进一步整合资源、健全体系、改善环境，提升城乡社区治理水平。完成水毁工程修复、乡镇防汛指挥图编制，发展智慧气象，提升防汛防台能力。健全安全生产责任体系，推进重点行业、重点领域专项整治，坚决遏制较大以上安全生产事故。以“四个最严”深化“餐桌污染”治理，打造食品安全名城。做好防震减灾工作。

同时，加强国防教育、国防动员、民兵预备役、人防、海防和双拥共建，推动军民融合深度发展。做好第四次全国经济普查工作。全面贯彻党的宗教工作基本方针，帮扶少数民族乡村加快发展。激发社会组织活力，支持工会、共青团、妇联、科协、红十字会等人民团体发挥更大作用。

三、加快建设人民满意的服务型政府

新时代开启新征程、续写新篇章。我们要不忘初心，牢记使命，不驰于空想，不骛于虚声，奋力开创政府工作新局面。

以新思想引领新征程。要用习近平新时代中国特色社会主义思想武装头脑，坚决维护以习近平同志为核心的党中央权威和集中统一领导，增强“四个意识”，坚定“四个自信”，扎实开展“不忘初心、牢记使命”主题教育，自觉做共产主义远大理想和中国特色社会主义共同理想的坚定信仰者和忠实实践者。

以新状态展现新作为。担当发展之责、为民之责，既要政治过硬，也要本领高强。要加强学习和实践，提升“八大本领”，培育专业能力，切实增强广大干部的创造力和执行力。加大正向激励，健全容错纠错机制，为苦干实干、不谋私利的干部撑腰鼓劲。以造福人民为最大政绩，精准查补民生短板，努力解决好“四难一差”问题，让群众少点操心事、烦心事，多点获得感、幸福感。大兴调查研究之风，坚持马上就办、真抓实干，以钉钉子精神，一步一个脚印，踏踏实实干好工作。

以新机制激发新活力。大胆解放思想，大胆打破常规，以“生态+”模式引领城市转型、产业转型，以试点试验推动机制变革、制度创新，向“机制活”要空间、要活力、要效益。坚决除繁苛之弊、施公平之策、开便利之门，下大力气简政便民，毫不手软地管一管难办的事、治一治难缠的人，让群众和企业办事不折腾、不闹心、不费劲。亲近不逾矩、清廉不懈怠，充分体现权利平等、机会平等、规则平等，努力构建“亲”、“清”新型政商关系，让营商环境更宽松、更优质、更洁净。

以新标尺塑造新形象。坚定不移推进全面从严治党，严格执行《准则》、《条例》，严格执行中央八项规定实施细则和省、市实施办法，进一步纠正“四风”、加强作风建设。织密扎紧制度笼子，管好公务人员、公共资源、公共权力、公共资金。深化政务“五公开”，让权力在阳光下运行。加强审计监督，支持配合国家监察体制改革。深化机关效能建设，坚决纠正不作为、慢作为、乱作为，坚决查处庸政、懒政、怠政，努力实现干部清正、政府清廉、政治清明。

各位代表！漳州加快发展其时已至，干事创业其势已成！让我们紧密团结在以习近平同志为核心的党中央周围，在省委、省政府和市委的坚强领导下，埋头苦干、开拓进取、攻坚克难，为谱写新时代富美新漳州建设的精彩篇章作出更大贡献！
